# Human Herpesviruses 6 and 7 and Central Nervous System Infection in Children^1^

**DOI:** 10.3201/eid1008.030788

**Published:** 2004-08

**Authors:** Asad Ansari, Shaobing Li, Mark J. Abzug, Adriana Weinberg

**Affiliations:** * University of Colorado School of Medicine, Denver, Colorado, USA;; † The Children's Hospital, Denver, Colorado, USA; 1 This study was presented in part at the Pediatric Academic Societies' Annual Meeting, Baltimore, Maryland, May 2002.; 2 Current affiliation: Avera Regional Hospital, Sioux Falls, South Dakota, USA.

**Keywords:** human herpesvirus 6, human herpesvirus 7, childhood meningitis, childhood encephalitis, childhood CNS disorders, research

## Abstract

The role and frequency of human herpesviruses (HHV)-6 and -7 in central nervous system (CNS) diseases of children are unclear. Cerebrospinal fluid samples from 245 pediatric patients (median age 43 days), submitted for evaluations of possible sepsis or of neurologic symptoms, were tested for HHV-6 and HHV-7 DNA by polymerase chain reaction. HHV-6 DNA was found in 3 of 245 samples, and HHV-7 was found in 0 of 245 samples. The three patients with HHV-6 DNA were <2 months of age. HHV-6 was likely pathogenic in two patients with meningitis who lacked evidence of another microbiologic cause. HHV-6 and HHV-7 are uncommon causes of CNS infection in children. HHV-6 may occasionally cause meningitis in young infants.

## Abstract 2

Human herpesviruses 6 and 7 were infrequently found in cerebrospinal fluid of children with central nervous system infection.

Human herpesvirus (HHV)-6 and HHV-7 are ubiquitous T-lymphotropic viruses that infect most humans. Infections with either agent occur primarily during childhood. Seroprevalence of HHV-6 reaches >80% in children >2 years (1–3). Antibody prevalence for HHV-7 reaches 75% in 3- to 6-year-old children and 98% in adults (3–5). HHV-6 and HHH-7 have been associated with a variety of clinical manifestations, including fever, rash, and seizures (6–10). Immunocompromised hosts, particularly transplant recipients, are at increased risk for symptomatic primary or reactivation disease associated with HHV-6 or HHV-7 (11–13).

The role of HHV-6 and 7 in central nervous system (CNS) disease is an area of ongoing investigation. The range of CNS manifestations ascribed to these viruses includes asymptomatic infection, febrile convulsions, seizure disorders, meningitis, meningoencephalitis, facial palsy, vestibular neuritis, demyelinating disorders, hemiplegia, and, rarely, fatal encephalitis (14–18). Investigators have been unable to culture HHV-6 or HHV-7 from cerebrospinal fluid (CSF) (14). However, HHV-6 and HHV-7 DNA have been detected in CSF and other body fluids by polymerase chain reaction (PCR), which implicates these viruses in neurologic disorders. HHV-6 DNA was identified in CSF of 14.8% of children evaluated for fever, sepsis, or seizures, with higher prevalence found among children with seizures (16). HHV-6 DNA was also detected in CSF of 70% to 90% of children who had neurologic symptoms during their primary HHV-6 infection, with a disproportionate association with recurrent febrile seizures (17). In a case-control study, HHV-6 DNA was found in CSF of 23% of patients who received an allogeneic bone marrow transplant who had CNS symptoms; it was found in <1% of patients with hematologic malignancies without neurologic symptoms (18). Other investigators have found a much lower prevalence (0%–4%) of HHV-6 DNA in CSF of AIDS patients with neurologic symptoms and in CSF of children with febrile seizures (19,20). Similarly, although HHV-7 DNA has been detected in CSF of as many as 8.8%–14% of children with neurologic symptoms (21,22), other studies have found a lower prevalence (0%–2%) in CSF of AIDS patients with neurologic symptoms and in children with febrile seizures (19,20).

Because of the conflicting results in the medical literature, the frequency at which HHV-6 and HHV-7 are associated with neurologic disease is unclear. The goal of this study was to further define the role of HHV-6 and HHV-7 as causes of CNS disease in children.

## Materials and Methods

### Study Design

The study, approved by the University of Colorado Multiple Institutional Review Board, was conducted with all CSF clinical samples from pediatric patients submitted for herpes simplex virus (HSV) PCR to the Clinical Virology Laboratory at the University of Colorado from December 1998 through February 2000. When multiple specimens were submitted for one patient, only the first one was tested. Specimens positive for other microorganisms were not excluded. Peripheral blood specimens from these patients were not available to study. Information regarding demographics, clinical manifestations, diagnostic studies, management, discharge diagnosis, and outcome was gathered by retrospective chart review for patients seen at The Children's Hospital, Denver. CNS diagnoses and classification of seizures were based on the assessments of the primary treating physicians.

### Definitions

Infectious and postinfectious encephalitis were defined as the presence of encephalopathy or focal neurologic abnormalities, an abnormal CSF profile but negative CSF microbiologic studies, and a history or serologic result consistent with a current or preceding acute infectious illness. CSF pleocytosis was defined as >25 leukocytes x 10^6^/L for preterm neonates, >22 leukocytes x 10^6^/L for term neonates, and >7 leukocytes x 10^6^/L for all other patients. Infections not involving the CNS were classified as other infections.

### HHV-6 PCR

HHV-6 PCR was performed (23) with the following primers and probes (24): 5´ AAG CTT GCA CAA TGC CAA AAA ACA G (17627–17603), 5´ AAC TGT CTG ACT GGC AAA AAC TTT T (17405–17429), and 5´ AAC TGT CTG GCA AAA ACT TTT (17516–17492). DNA was extracted from 50-µL aliquots of CSF previously stored at –70°C with Chelex purification matrix (Bio-Rad Laboratories, Inc, Hercules, CA). PCR was carried out in 50-µL mixtures containing 20 µL of extracted DNA, 1.25 units of PfuI (Stratagene, La Jolla, CA), 200 µmol/L of each of four deoxynucleoside triphosphates, and 0.5 µmol/L of each primer in PfuI buffer (Stratagene). The samples were amplified in duplicate for 45 cycles. The amplified DNA was separated according to molecular weight by using 3% agarose gel electrophoresis and transferred to nylon membranes. The identity of the DNA band (223 bp) was confirmed by detection with a digoxigenin-conjugated probe, antidigoxigenin antibody conjugated to alkaline phosphatase (Boehringer Mannheim Biochemicals, Indianopolis, IN), and chemiluminescent substrate (Tropix, Bedford, MA). Each patient sample was run in duplicate. Each assay included two negative controls (water) and two positive controls (100 genomic copies of HHV-6 DNA/reaction tube). The tests were considered valid if the controls yielded the expected results, and a specimen was considered positive if both replicates tested positive. Specimens with discordant replicates were reanalyzed in an independent run and considered positive if >50% of replicates gave positive results. The ability to detect HHV-6 DNA after at least four freeze-thaw cycles remained intact.

The analytical sensitivity of HHV-6 PCR was 8–10 copies of genomic DNA per reaction tube, as determined by serial dilutions of a sample with known DNA copy number. To rule out the presence of inhibitors in the extracted DNA, 20 HSV-negative CSF samples were spiked with the equivalent of 2 HSV DNA copies per PCR reaction tube. All samples yielded a positive result for HSV, whereas the unspiked specimens remained HSV-negative. The ability to detect HHV-6 strains from different geographic regions was confirmed by using 14 HHV-6–containing specimens obtained from diverse laboratories with recognized expertise in diagnosing HHV-6 infections as well as our laboratory. No cross-reactivity occurred with other DNA viruses, including other herpesviruses, parvovirus, adenovirus, and polyomavirus, thereby establishing the specificity of the HHV-6 PCR.

### HHV-7 PCR

HHV-7 PCR was performed by using the methods described for HHV-6 with modified sample preparation. The primers were 5´ TAT CCC AGC TGT TTT CAT ATA GTA AC and 5´ GCC TTG CGG TAG CAC TAG ATT TTT TG, and the probe was 5´ AGA ATT CTG TAC CCA TGG GCA CAT TTG TAC (25; GenBank accession no. L03525). The PCR product was 186 bp long. The assay included two negative controls (water) and two positive controls (100 genomic copies of HHV-7 DNA per reaction tube).

The sensitivity of HHV-7 PCR was 8–10 copies of DNA per reaction tube, as determined by detecting HHV-7 DNA from samples extracted from infected cells. Specimens were prepared by boiling aliquots of nonhemorrhagic CSF for 55 s. If CSF contained blood, DNA was extracted with Qiagen DNA extraction kit (Qiagen, Valencia, CA). Testing with control viral DNA indicated that boiling did not affect the sensitivity of the PCR. The presence of inhibitors was ruled out by spiking 20 HSV-negative CSF samples with the equivalent of two HSV DNA copies per reaction tube and showing that all spiked samples became HSV-positive, whereas unspiked samples remained negative. The ability to detect HHV-7 DNA after at least three freeze-thaw cycles remained intact.

## Results

### Study Population

CSF samples obtained from 245 patients were tested for HHV-6 and HHV-7 DNA by PCR. Clinical data were available for the 218 patients hospitalized at The Children's Hospital, Denver (Table 1). The median age was 43 days; 68% were <2 years of age; 13% were 2–6 years, and 19% >6 years. Fever occurred in 56% of patients, rash in 11%, and seizures in 25%. CNS disorder diagnoses at discharge included meningitis in 61 patients (enteroviral, 21; bacterial, 4; aseptic/indeterminate etiology, 36), encephalitis in 21 patients (HSV, 4; acute disseminated encephalomyelitis, 4; infectious/postinfectious, 13), febrile seizures in 7 patients, and seizure disorder in 29 patients. A non-CNS disease had been diagnosed in the remaining 100 patients, which included a diagnosis of other infections in 60. Nineteen patients were immunocompromised (leukemia, 6; posttransplant, 3; premature, 3; HIV, 2; others, 5), of whom 1 had a febrile seizure, 1 had encephalitis, 2 had meningitis, and 5 had other infections. CSF pleocytosis was found in 90 (43%) of 209 patients for whom data were available.

**Table 1:** Characteristics of the study population with available clinical data^a^

Characteristic	No. patients (%)
Demographic	
Age: median (range)	43 days (0–5,948 days)
<2 y	148 (68)
2–6 y	28 (13)
>6 y	42 (19)
Male:female	1.1:1
Clinical and laboratory	
Fever	121 (56)
Rash	24 (11)
Seizures	53 (25)
Seizure disorder	29 (55)
Febrile	7 (13)
Metabolic	5 (9)
Infectious/postinfectious	5 (9)
Intracranial bleed	4 (8)
Hypoxia	3 (6)
Meningitis	61 (28)
Encephalitis	21 (10)
CSF pleocytosis	90 (43)
Immunocompromised	19 (9)

^a^N = 218; CSF, cerebrospinal fluid.

### HHV-6 Infections of CNS

PCR identified HHV-6 DNA in CSF of 3 of 245 patients (Table 2). Patient 1, a 5-day-old full-term male infant of a diabetic mother, had a generalized seizure on the day he was born. His seizure was attributed to hypoglycemia because his concurrent glucose level was 0 mmol/L. On day 5 of life, a fever of 38.3ºC developed for which he underwent a complete workup for sepsis. Results of a physical examination were normal with no signs or symptoms of neurologic dysfunction. His blood count was remarkable for a leukocyte count of 8,600 x 10^6^/L (46% neutrophils and 21% bands) and a platelet count of 40,000 x 10^6^/L. His CSF showed 40 leukocytes x 10^6^/L (20% neutrophils, 9% bands, 31% lymphocytes, 33% monocytes, and 6% macrophages), 4,110 erythrocytes x 10^6^/L, protein 169 g x 10^-2^/L, and glucose 2.3 mmol/L. CSF HSV PCR, viral culture, and bacterial cultures of CSF and blood and urine were negative. Diagnosis at discharge was aseptic meningitis. The patient recovered spontaneously without sequelae.

**Table 2:** Characteristics of patients with HHV-6 DNA detected in cerebrospinal fluid^a^

Patient	Age (d)	Temperature maximum (°C)	Rash	Seizure	CSF leukocyte count (cells/µL)	Outcome	HHV-6 directed treatment
1	5	38.3	No	Yes^b^	40	Alive	None
2	38	38.4	Yes	No	19	Alive	None
3	10	38.7	No	No	1	Deceased	None

^a^CSF, cerebrospinal fluid; HHV-6, human herpesvirus 6. ^b^Infant of a diabetic mother who had a seizure on the day he was born. His serum glucose level was 0. Lumbar puncture was performed for a new fever on day 5 of life.

Patient 2 was a 38-day-old full-term female infant who had a fever of 39.2ºC and irritability. The physical examination showed a few vesicular lesions on the tonsillar pillar as well as a diffuse, erythematous, macular blanching rash. CSF studies found 19 leukocytes x 10^6^/L (34% lymphocytes, 12% monocytes, and 55% macrophages), 1 erythrocyte x 10^6^/L, protein 48 g x 10^-2^/L, and glucose 2.3 mmol/L. PCRs of CSF for HSV and enterovirus and bacterial cultures of CSF, blood, and urine were negative. She recovered spontaneously without sequalae. Her discharge diagnosis was aseptic meningitis.

Patient 3 was a male infant of 24 weeks' gestation with respiratory distress syndrome. A blood culture was taken on day 10 of life because of worsening respiratory distress; results were positive for *Candida*. A lumbar puncture was performed when the blood grew *Candida*. The patient did not have any neurologic symptoms or signs. CSF had 1 leukocyte x 10^6^/L, 1,780 erthrocytes x 10^6^/L, protein 105 g x 10^-2^/L, and glucose 8.1 mmol/L. CSF studies, including PCRs for HSV and enterovirus, as well as bacterial, viral, and fungal cultures, were negative. He subsequently died of the complications of prematurity and candidal sepsis, which were unrelated to the CNS.

All 3 were <2 months of age, and 2 had CSF pleocytosis. Overall, HHV-6 DNA was detected in 2 (2.2%) of 90 specimens from patients with pleocytosis and in 2 (3.3%) of 61 patients with a discharge diagnosis of meningitis. HHV-6 DNA was not found in any patients who had encephalitis, febrile seizures, or a seizure disorder. None of the 19 immunocompromised patients had HHV-6 DNA in CSF.

### HHV-7 Infections of CNS

HHV-7 DNA was not found in any of the 245 CSF samples tested by PCR. Spiking random samples with HHV-7 DNA generated positive PCR results.

## Discussion

HHV-6 DNA was uncommon in CSF of children (1.2%), and HHV-7 DNA was not detected in any CSF specimen in this study. HHV-6 DNA was found in a small percentage of meningitis patients (3.3%), but neither HHV-6 nor HHV-7 DNA was detected in samples from any patients with encephalitis, febrile seizures, or seizure disorders. Our limited detection of HHV-6 and HHV-7 contrasts with results of other studies, such as the report of Caserta et al. (16), in which HHV-6 DNA was found in CSF of 14.8% of children evaluated for fever, sepsis, or seizures, and studies by Pohl-Koppe et al. (21) and Yoshikawa et al. (22), in which HHV-7 DNA was found in CSF of 8.8% to 14% of children with neurologic symptoms. Our findings may have differed from previous reports for the following reasons: 1) differences in PCR sensitivity; 2) differences in degree of intactness of DNA in specimens that were thawed after being frozen for prolonged periods; 3) geographic variation; or 4) differences in patient populations and, particularly, differences in age groups.

Technical laboratory problems were unlikely. Our assays are as sensitive or more sensitive than those previously reported that use plasmid-encoded DNA, and we detected a panel of samples identified by other laboratories, which use different primers or probe sets.. However, these latter experiments were limited in number and could not completely rule out sensitivity differences related to the primers and probe sets. The presence of inhibitors was ruled out by spiking experiments. We tested the effect of freezing and thawing by subjecting several samples to three to four freeze-thaw cycles and found good preservation of both HHV-6 and HHV-7 DNA. Also, the PCR method used in this study detected cytomegalovirus DNA in CSF specimens stored at –70°C for up to 6 years (23). Yoshikawa et al. (22), who reported a 16% incidence of HHV-6 and HHV-7 in their pediatric patients with neurologic disorders, detected the viral DNA in the CSF cell pellet, but not in the liquid phase. Other investigators, however, who found a high incidence of HHV-6 and HHV-7 in pediatric patients with neurologic disorders (16,21), recovered viral DNA from liquid CSF.

Our results are not likely to be biased by the study population. Although lower seroprevalence rates have been found in some regions of the world, for example, Morocco (20% for HHV-6) and northern Japan (44% for HHV-7) (5,26), no evidence has been found of low seroprevalence of HHV-6 and HHV-7 infections in specific regions of the United States. Also, the demographic characteristics of our study population were similar to those of the 487 children described by Caserta et al. (16), including a median age similar to the median age of 1.5 months (range 0.1–36) in the latter series (16). The populations evaluated by Yoshikawa et al. (22) and Pohl-Koppe et al. (21) were somewhat older, with mean ages of 6 and 2.8–6 years, respectively. However, the number of patients >2 years of age included in our study population was similar to the numbers of participants enrolled in these two studies (43 and 68, respectively). Therefore, age differences do not appear to explain the discrepant findings.

The clinical features of patients 1 and 2 and the absence of another identified infectious agent suggest that HHV-6 may have been the cause of fever, pleocytosis, and the clinical aseptic meningitis diagnosed in these two infants. Other studies have not found an association between HHV-6 DNA in CSF and CSF pleocytosis. However, we note that the CSF of patient 1 was contaminated by blood. Therefore, the presence and magnitude of pleocytosis are difficult to state with certainty; moreover, we cannot be certain whether HHV-6 DNA was truly present in CSF or derived from contamination with peripheral blood.

In patient 3, the identification of a well-established pathogen and the normal results of neurologic and CSF examinations suggest that HHV-6 DNA in the CSF may have represented an asymptomatic infection or contamination with peripheral blood. The cases of HHV-6 infection of CNS occurred in infants <2 months of age, and two occurred in the early neonatal period. Infections in newborns have been previously described. Although horizontal transmission through saliva is postulated to be the most common method of transmission of HHV-6 and HHV-7 (27–29), infection in neonates as well as detection of HHV-6 DNA in aborted fetuses' cord blood and in the female genital tract suggest that transplacental or perinatal transmission is possible (30–33).

Our study included 29 patients with CNS disorders caused by a known pathogen other than HHV-6 and HHV-7 (enterovirus, herpes simplex virus, bacterial pathogen); none of these patients had HHV-6 or HHV-7 DNA in CSF. This finding suggests that HHV-6 or HHV-7 DNA is not frequently detected in CSF in association with other CNS infections, as might be expected if these viruses established latency in CNS and reactivated nonspecifically with other pathogens. Conversely, this finding supports the idea that detecting HHV-6 or HHV-7 DNA in CSF of patients with neurologic disorders is consistent with a pathogenic role. Our study population included a modest number of immunocompromised patients of whom a small subset had neurologic diagnoses. These numbers are insufficient to ascertain the roles of HHV-6 and HHV-7 in neurologic disease in immunodeficient patients.

In conclusion, our data indicate that HHV-6 and HHV-7 are uncommon causes of CNS infection in children. HHV-6 may occasionally cause meningitis in young infants. Further studies are required to define the role of HHV-6 and HHV-7 in neurologic disorders in immunocompromised hosts.
